# Metabolic equivalents of task are confounded by adiposity, which disturbs objective measurement of physical activity

**DOI:** 10.3389/fphys.2015.00226

**Published:** 2015-08-11

**Authors:** Tuomo T. Tompuri

**Affiliations:** ^1^Department of Clinical Physiology and Nuclear Medicine, Kuopio University HospitalKuopio, Finland; ^2^Institute of Biomedicine/Physiology, School of Medicine, University of Eastern FinlandKuopio, Finland

**Keywords:** adiposity, energy expenditure, intensity, lean mass, metabolic equivalent, MET, PAEE, physical activity

## Abstract

Physical activity refers any bodily movements produced by skeletal muscles that expends energy. Hence the amount and the intensity of physical activity can be assessed by energy expenditure. Metabolic equivalents of task (MET) are multiplies of the resting metabolism reflecting metabolic rate during exercise. The standard MET is defined as 3.5 ml/min/kg. However, the expression of energy expenditure by body weight to normalize the size differences between subjects causes analytical hazards: scaling by body weight does not have a physiological, mathematical, or physical rationale. This review demonstrates by examples that false methodology may cause paradoxical observations if physical activity would be assessed by body weight scaled values such as standard METs. While standard METs are confounded by adiposity, lean mass proportional measures of energy expenditure would enable a more truthful choice to assess physical activity. While physical activity as a behavior and cardiorespiratory fitness or adiposity as a state represents major determinants of public health, specific measurements of health determinants must be understood to enable a truthful evaluation of the interactions and their independent role as a health predictor.

## Introduction

This review discusses critically the metabolic equivalent of task (MET) (Weir, 1949; Jetté et al., 1990), and its applicability to measure energy expenditure or to assess amount and intensity of physical activity. Physical activity is a major public health determinant (Lim et al., 2012) and physical activity introduces public health promoting potential. Physical activity research consists also behavioral aspect, which has been taken into account in elaborate review by Hills et al. (2014), but from physiological point of view specific and truthful measurement is needed to assess physical activity related health interactions. While physiologists are familiar with physiological relevance and potential problems of body size differences related scaling (Tanner, 1949; Weir, 1949; Hoppeler and Weibel, 2005; Lorenzo and Babb, 2012; Tompuri et al., 2014a), there are physiological procedures which have been adapted to be used at the population level in health sciences. For example scaling by body weight or METs has often been used when aiming to assess physical activity (Strath et al., 2001, 2013; Brage et al., 2004, 2005; Corder et al., 2005; Crouter et al., 2008; US Department, 2008; Warren et al., 2010; World Health Organization, 2010). This paper enlightens the background of the MET as well as rational body size-related scaling, and demonstrates how methodological confounding may affect physical activity assessment, which in turn may bias further analytical conclusions.

## Physiological basis for measurement of physical activity

The definition of the physical activity as “*any bodily movements produced by skeletal muscles that result in energy expenditure*” includes a rationale why energy expenditure can be used to assess physical activity (Caspersen et al., 1985). Physical activity determines the most variable portion of the energy expenditure; moreover, the rate of the energy expenditure is directly linked to the intensity of the physical activity (Strath et al., 2013). While oxygen uptake has equivalency with energy expenditure during rest and physical activity (Weir, 1949; Dennis and Noakes, 1998), muscle blood flow is closely related to the oxygen demand of the exercising muscles (Andersen and Saltin, 1984). Furthermore, the heart rate reflects the level of the oxygen supply (Stringer et al., 1997) and energy metabolism regardless of the type of dynamic exercise (Strath et al., 2000; Achten and Jeukendrup, 2003). Therefore, ambulatory heart rate recording can be used to assess long-term energy expenditure. As compared with other methods to assess physical activity, such as questionnaires or movement sensors, an objective measurement of the energy expenditure during dynamic exercise by ambulatory heart rate recording has value in defining the intensity of physical activity especially at greater intensities (Warren et al., 2010).

The energy cost of a physical activity can be expressed by the METs that reflect the metabolic rate (Weir, 1949; Jetté et al., 1990). As a physiological reference for a man who weighs 70 kg, one MET has been defined as 40 kcal/square meter of the body surface area^*^h. However, its derivative 1 kcal/kg^*^h, which refers an oxygen uptake of 250 ml/min, corresponds to the standard MET expression that is scaled by body weight i.e., 3.5 ml/min/kg (Jetté et al., 1990; Byrne et al., 2005). This value reflects the resting metabolism during quiet sitting. During physical activity, multiples of the resting metabolisms refer the metabolic rate and aims to standardize the energy cost greater than resting metabolism (Warren et al., 2010). Therefore, standard MET vs. time integral at daily level should reflect the amount and intensity of physical activity.

Conversely, the standard expression of one MET has been criticized because resting metabolism varies according to the physiological state (Saris et al., 2003; Byrne et al., 2005; Harrell et al., 2005; Kozey et al., 2010; Wilms et al., 2014). The size of the individual is a major determinant of the basal metabolism (Kleiber, 1947; Ravussin et al., 1986) as well as maximal workload and oxygen uptake (Jensen et al., 2001; Wasserman et al., 2005). Therefore, to enable comparison between individuals the absolute values should be scaled by body size (Jensen et al., 2001; Wasserman et al., 2005; Strath et al., 2013). Already in 1883, metabolism was discovered to be proportional to the surface of the body (Rubner, 1883), and in 1949, while determining the ratio between oxygen uptake and energy expenditure, Weir proposed that the metabolic rate should be expressed by the body's surface area instead of body weight (Weir, 1949). On other hand, exercise testing has originally been used among endurance athletes who are lean subjects, and in competitive sports power produced per body weight as an indicator of functional capacity matters more than measurement of the cardiorespiratory capacity in the physiological context (Lee et al., 2002). However, scaling by body weight is widely used at the population level in health sciences and epidemiology.

## Rationale of the body size differences related scaling

Simple measures, such as height or weight, can easily be used to assess body size, but the problem is that these measures may not be able to distinguish the relevant physiological differences between subjects. The metabolic size matters more as compared to dimensional differences. While the body weight includes fat mass, energy metabolism is related to lean mass. Therefore, scaling by body weight can cause a statistical problem due to “mathematical coupling” with adiposity (Firebaugh and Gibbs, 1985). Body surface area (DuBois and DuBois, 1916), as a fractal and indirect indicator (Heaf, 2007) of body size, cannot identify metabolically relevant lean mass content in the way modern body composition measurements do (Fosbøl and Zerahn, 2015). Physiological (Goran et al., 2000; Tompuri et al., 2014a), mathematical (Firebaugh and Gibbs, 1985), or physical rationales for scaling oxygen uptake or energy expenditure by body weight cannot be found.

Basal metabolism is strongly related to lean tissue (Ravussin et al., 1986), and physical activity-related energy expenditure is produced by skeletal muscles (Caspersen et al., 1985; Hoppeler and Weibel, 2000). The skeletal muscle mass *per se* is a major determinant for increased metabolism during exercise (Cooper et al., 1984; Tipton and Franklin, 2006) and the absolute maximal oxygen uptake (Turley and Wilmore, 1997; LeMura et al., 2001). Therefore, scaling by lean mass is a physiologically rational method to perform body size related normalization (Osman et al., 2000; American Thoracic Society, American College of Chest Physicians, 2003; Krachler et al., 2014). Correspondingly, while fat tissue is energy metabolically inactive during exercise (Andersen and Saltin, 1984; Goran et al., 2000), fat mass represents most inter-individually variable compartment of the body (Fomon et al., 1982; Bazzocchi et al., 2013). Therefore, scaling by body weight has been criticized (Tanner, 1949; Lorenzo and Babb, 2012). Hence, while energy expenditure has equivalency with oxygen uptake, body size-related normalization of the energy expenditure should be done by lean mass as in case of the oxygen uptake.

**Figure 1:**
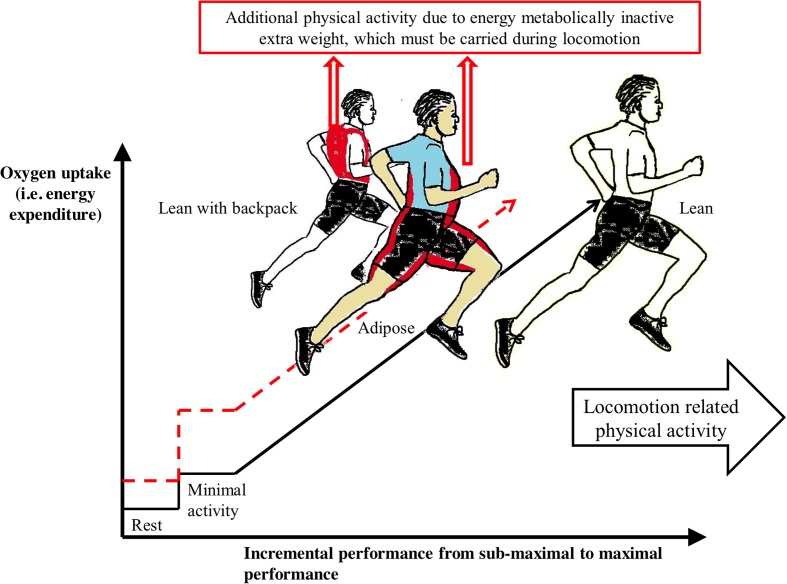
**Additional work by extra load (marked by red color and dashed line) due to adiposity or due to backpack limits maximal performance and increases sub-maximal performance related energy cost during locomotion related exercise**. Maximal oxygen uptake referring maximal energy expenditure is similar between subjects as well as lean mass. Increase of oxygen uptake by exercise does not differ between lean (solid line), adipose and lean with backpack subjects (Wasserman et al., 2005). Dimensions of the curves are normative and directional.

Physically fat mass represents a load that must be carried during physical activity. According to Newton's second law of motion, mass, such as extra mass by adiposity, increases the force. Therefore, adiposity increases total work of exercise during locomotion-related physical activity (Cureton and Sparling, 1980). Respectively, body fat excess increases basal metabolism because adiposity also increases the amount of lean mass (Wasserman et al., 2005; Heymsfield et al., 2014). However, body fat *per se* does not affect the slope of the increase in oxygen uptake with an increase in the external workload or the maximal aerobic capacity (Goran et al., 2000; Wasserman et al., 2005) (Figure 1). Interestingly, the observation that energy expenditure during physical activity increases as body weight increases has been assumed to justify the use of body weight in the scaling of energy expenditure (Strath et al., 2013). However, this assumption lacks physiological and physical rationale. While exercise, such as movement from one place to another, is one task causing energy cost, the subject must simultaneously carry his excess fat mass, which is an additional task. Both tasks are physical activities, which are produced by skeletal muscles. Therefore, scaling to normalize body size differences should be performed by metabolic size, i.e., by lean mass.

## Examples of methodological hazards encountered when using standard MET

Unfortunately the current review is not only theoretical speculation, but has practical relevance. Standard METs have been used in many original methodological publications of physical activity (Strath et al., 2001; Brage et al., 2004, 2005; Corder et al., 2005; Crouter et al., 2008), and these publications have been cited in reviews (Warren et al., 2010; Strath et al., 2013). Furthermore, body weight scaled METs have been used in recommendations for physical activity measurement in North America (US Department, 2008) and in global recommendations on physical activity by the World Health Organization (World Health Organization, 2010).

While measuring physical activity, the potential problems due to body weight scaled METs may be clarified through simple examples using imaginary subjects: (a) a lean subject, (b) an obese subject, and (c) a lean subject with a backpack representing an extra load to be carried (Figure 1). To make this example easier to understand, these subjects have identical lean mass, absolute maximal oxygen uptake and maximal heart rate. These examples demonstrate how extra weight carried during similar sub-maximal or maximal performance effects on alternative indicators of physical activity, such as (1) absolute energy expenditure, (2) lean mass proportional energy expenditure, (3) relative intensity of physical activity and (4) body weight-scaled energy expenditure, i.e., standard METs.

### Absolute energy expenditure

A more obese subject has a greater absolute energy cost as compared with a leaner subject when they are moving at a similar sub-maximal speed because excess body fat is an extra load that must be carried (Figure 1). This agrees with Newton's second law of motion. While the ratio of work and oxygen cost due to locomotion in adipose is similar to leaner subject, the additional energy cost is caused by the greater work because of extra load due to the additional fat mass. The comparison is similar between lean subjects with and without the extra load.

In maximal exercise, the subjects would have similar maximal absolute energy expenditure because they have similar maximal absolute oxygen uptake [ml/min]. However, a lean subject would be able to achieve a greater level of external performance than the obese subject because the lean subject uses less of the physiological reserve to carry his body weight, as observed by Cureton and Sparling (1980) (Figure 1).

### Lean mass proportional energy expenditure

When using scaling energy expenditure by lean mass, the same exercise would introduce a similar lean mass proportional energy cost due to exercise performance (Wasserman et al., 2005) (Figure 1). However, additional work due to the extra load increases the lean mass proportional oxygen uptake and energy expenditure as compared to the situation without extra load.

Maximal levels of the lean mass proportional energy expenditures would be similar, but subjects with a backpack or greater fat mass would be unable to perform similar locomotion related maximal performance as the lean subject without an extra load (Cureton and Sparling, 1980).

### Relative intensity of physical activity

The relative intensity of physical activity refers to the percentage of the maximal oxygen uptake or percentage of maximal heart rate. As compared to a lean subject with a similar absolute maximal oxygen consumption, an obese subject would experience a greater relative intensity of exercise at any given sub-maximal performance level because excess fat mass must be carried during locomotion (Cureton and Sparling, 1980). Correspondingly, if the lean subject carries a backpack, he would achieve a greater intensity at any sub-maximal performance level as compared to the situation without any extra load.

Maximal energy expenditure, maximal oxygen uptake or maximal heart rate can be achieved with or without extra load. However, the extra load diminishes the maximal external performance, such as running speed (Cureton and Sparling, 1980) (Figure 1), which also agrees with Newton's second law of motion.

### Energy expenditure by body weight scaled METs

If the energy expenditure were expressed proportionally to body weight, i.e., using standard METs, the obese subject would have a lower level of physical activity than a lean subject with the same absolute energy expenditure. If the subjects walk at the same pace, the absolute or lean mass proportional energy cost as well as intensity relative to maximal oxygen intake or heart rate of the adipose subject would be greater. This false observation results because body weight includes confounding by adiposity when comparing subjects. The extra load caused by the fat mass does not in and of itself impair aerobic capacity or maximal energy expenditure (Goran et al., 2000; Tompuri et al., 2014a), even though the extra load impairs physiological performance (Cureton and Sparling, 1980) and increases energy cost as compared to lean subjects without the extra load.

## Summary of practical and analytical relevance

It is paradoxical that a subject who has a greater absolute energy expenditure and a greater relative intensity during similar performance may be classified as less physically active if using standard METs. Moreover, it is interesting that the original definition of the MET (Weir, 1949) was aware of the potential problems caused by body size normalization by body weight. Therefore, the use of by body weight-scaled standard METs should be avoided when assessing physical activity-related energy expenditure.

When measuring physical activity, it is important to measure essential dimensions instead of irrelevant confounders (Warren et al., 2010). Similarly, it is important to recognize the metabolically relevant size when performing body size-related normalization (Goran et al., 2000; Hoppeler and Weibel, 2000; Krachler et al., 2014). However, although scaling by body weight represents physiologically historic burden, reporting by METs represents a kind of state of practice in objective measurement of physical activity, maybe because the MET is a simple measure (Jetté et al., 1990; Hills et al., 2014). For example as a well-known method METs and scaling by body weight are often used in studies considering survival rates (Holick et al., 2008) or applied physiology (Turzyniecka et al., 2010). However, because of scaling confounded by adiposity there may be undetected interactions between relevant measures. The body lean mass vs. fat mass ratio declines with aging (Bazzocchi et al., 2013) and would introduce inevitably decline in physical activity if using standard METs. Similarly, cardiovascular decline (Santulli et al., 2013) and impairment in insulin sensitivity (Santulli et al., 2012) are prominent features with aging and are also affected by adiposity, which should be taken into account analytically, especially if analyzing with METs.

Adiposity is a major public health risk (Lim et al., 2012), and confounding by adiposity refers to a situation whereby body weight-scaled measures reflects adiposity instead of cardiorespiratory fitness and physical activity *per se*. In general, as the prevalence of adiposity has increased at the population level (Vuorela et al., 2011), potential confounding by adiposity has become even more important. This causes an increased risk of false conclusions, if relevant residual confounding has not been detected when assessing interactions e.g., between physical activity, adiposity and cardiorespiratory fitness (Wong et al., 1999; Tompuri et al., 2014a,b; Wilms et al., 2014). For example, when using standard METs, confounding by adiposity may result in the biased conclusion that greater intensity and a greater amount of physical activity would be healthier *ad infinitum*, because more fit and leaner, i.e., healthier subjects, would be classified as engaging in increasingly intensive physical activity as compared to adipose and unfit subjects even if physical activity levels based on absolute energy expenditure were similar.

It is important to realize that statistical adjustment for adiposity may not completely eliminate problems related to residual confounding (Wong et al., 1999) introduced by standard METs. It has been observed that the standard MET as compared to individually measured resting metabolism disproportionally impacts subgroups of the population and causes analytical errors when assessing physical activity (Kozey et al., 2010). Theoretically individually measured resting metabolism instead of the standard MET would potentially diminish analytical problems (Byrne et al., 2005; Wilms et al., 2014), because similar scaling error would affect both resting metabolism and energy expenditure by exercise. On other hand, resting metabolic rate is a quite artificial measure (Hoppeler and Weibel, 2005), and also measurement errors will be multiplied along energy expenditure multiplies, which may cause analytical problems (Wong et al., 1999).

## Compendium

To understand physical activity as a behavior and cardiorespiratory fitness or adiposity as a state representing major determinants of public health, the specific measurements of these determinants must be understood to enable a truthful evaluation of their interactions and their independent role as a predictor of health outcomes.

A major advantage in the determination of energy expenditure is that different methods of measurement, such as movement counts by accelerometer and recordings of heart rate, can be combined, which may improve the accuracy of physical activity assessment over a broad range of intensity levels (Warren et al., 2010). Energy expenditure can be assessed also without standard METs. Whereas, oxygen uptake is equivalent to energy expenditure (Weir, 1949), interpretation of a maximal exercise performance with body composition measures (Tompuri et al., 2014a) depends on whether oxygen consumption is scaled by body weight or lean mass. Thus, we can conclude that scaling energy expenditure by lean mass would allow to avoid confounding by adiposity when comparing energy expenditure between subjects.

### Conflict of interest statement

The author declares that the research was conducted in the absence of any commercial or financial relationships that could be construed as a potential conflict of interest.
